# Physical activity promotion in the age of climate change

**DOI:** 10.12688/f1000research.23764.2

**Published:** 2020-11-20

**Authors:** Karim Abu-Omar, Peter Gelius, Sven Messing

**Affiliations:** 1 Department for Sport Science and Sport, Friedrich-Alexander-Universität Erlangen-Nürnberg (FAU), Erlangen, 91054, Germany

**Keywords:** physical activity, sport, active transport, exercise, training, recreation, climate change, climate action

## Abstract

The importance of the global climate crisis requires linking physical activity promotion and climate action. This article provides a first overview of interconnections between physical activity promotion and climate action, potential synergies and discrepancies, aiming to stimulate further discussion about this topic. The analysis is based on the World Health Organization’s Global Action Plan on Physical Activity 2018-2030 (GAPPA). The GAPPA covers five perspectives that are of particular relevance with respect to potential links with climate policy: (1) Infrastructures supporting active transport, (2) green spaces and recreational/exercise facilities, (3) exercise programs, (4) mass communication campaigns and mass participation events and (5) training of professionals. Our analysis demonstrates a considerable alignment between strategies for physical activity promotion and efforts for the reduction of greenhouse gas emissions. However, in some of the areas, this alignment could still be improved. Additionally, more climate-conscious policies, research and surveillance need to be developed in the field of physical activity promotion.

## Introduction

In recent years, there has been a new wave of recognition regarding the urgency of the global climate crisis. The Intergovernmental Panel on Climate Change (IPCC) has clearly stated that the next ten years will be crucial for limiting global warming to less than 1.5 degrees Celsius ^1^. Studies by the World Bank predict the devastating impact global warming will have on development of poor countries ^2^ and consequently on migration ^3^. The IPCC has also reported on the dramatic implications of climate change on land use ^4^. Nonetheless, the United Nations have vividly described the acute failure to curb global greenhouse gas emissions ^5^. In light of these sobering prospects, a number of American public health organizations have called on governments, business leaders and civil society to treat climate change as a “health emergency” ^6^. Also, there have been calls from scientists to engage in civil disobedience as a mean to put pressure on world leaders to step up efforts to combat the climate crisis ^7^.

Likewise, physical activity (PA) promotion has evolved significantly in the past decades and has asserted itself as a stand-alone public health issue ^8^. This development was triggered by a robust understanding of the role of PA for the prevention of non-communicable diseases ^9^ on the one hand and stubbornly high rates of insufficient physical activity in adolescents (81,0%) ^10^ and adults (27,5%) ^11^ on the other. The policy response has resulted, among others, in the PA Strategy for the World Health Organization (WHO) European Region 2016–2025 (EuroPAS; ^12^), the Global Action Plan on PA 2018–2030 (GAPPA; ^13^), the EU Council Recommendation on Health-Enhancing PA across Sectors ^14^, as well as numerous strategies, action plans and recommendations at the national level ^15– 17^.

But are efforts to promote PA in any way linked to the climate crisis, and if so, what are these links? Will the climate crisis dampen efforts for PA promotion or, to the contrary, are there potential synergies, with PA promotion potentially supporting efforts to combat climate change? What is the carbon footprint of different strategies currently employed or suggested to promote PA? What are potential upcoming research priorities in our field that relate to the climate crisis? These questions are highly relevant given the urgency of climate change and the need to prioritize limited resources. The global-level GAPPA acknowledges this by including a link to the United Nation’s Sustainable Development Goals (SDGs), including SDG 13 on climate action. However, the link between PA promotion and climate change plays a rather minor role in the GAPPA and, in our opinion, still needs to be further explored. This article is an attempt to provide a first overview of the available information and stimulate further discussion about these topics. It focuses mainly on climate change mitigation and the relation of rising temperatures and PA promotion.

## Interconnections between physical activity promotion and the climate crisis

The recent strategies developed by WHO to support national-level PA promotion provide a good overview of the most common action areas currently proposed. For example, the PA Strategy for the European Region ^12^ employs a life-course approach with four major action areas (leadership and coordination; children and adolescents; adults; older people; monitoring, surveillance, evaluation and research) and 14 specific objectives. The global-level GAPPA ^13^ builds extensively on this earlier strategy and has very similar key messages, but it uses a slightly different structure with four strategic objectives (active societies; active environments; active people; active systems) and 20 specific policy actions (see Table 1). The overview provided in the next sections is based on this most recent WHO policy document, taking a closer look at its different policy actions using five perspectives that bear particular relevance for climate change.

**Table 1.** Strategic objectives and recommended policy actions of the Global Action Plan on Physical Activity 2018–2030.

Strategic objectives	Policy actions (summary)
Create active societies	1.1. Implement best practice communication campaigns (…) to heighten awareness (…) of (…) the multiple health benefits of regular physical activity (…).
	1.2. Conduct national and community-based campaigns to enhance awareness (…) of (…) the social, economic, and environmental co-benefits of physical activity (…).
	1.3. Implement regular mass participation initiatives in public spaces (…).
	1.4. Strengthen pre- and in-service training of professionals, within and outside the health sector (…).
Create active environments	2.1. Strengthen the integration of urban and transport planning policies (…).
	2.2. Improve the level of service provided by walking and cycling network infrastructure (…).
	2.3. Accelerate implementation of policy actions to improve road safety (…).
	2.4. Strengthen access to good-quality public and green open spaces (…).
	2.5. Strengthen the policy, regulatory and design guidelines and frameworks (…) to promote (…) facilities (…) that are designed to enable occupants and visitors with diverse abilities to be physically active in and around the buildings.
Create active people	3.1. Strengthen provision of good-quality physical education (…).
	3.2. Implement and strengthen systems of patient assessment and counselling on increasing physical activity (…).
	3.3. Enhance provision of, and opportunities for, more physical activity programmes and promotion in parks and other natural environments (…).
	3.4. Enhance the provision of, and opportunities for, appropriately tailored programmes and services aimed at increasing physical activity (…) in older adults (…).
	3.5. Strengthen the development and implementation of programmes and services (…) to engage with (…) physical activity in the least active groups (…).
	3.6. Implement whole- of community initiatives (…).
Create active systems	4.1. Strengthen policy frameworks, leadership and governance systems (…) to support implementation of actions aimed at increasing physical activity (…).
	4.2. Enhance data systems and capabilities (…).
	4.3. Strengthen the national and institutional research and evaluation capacity and stimulate the application of digital technologies and innovation (…).
	4.4. Escalate advocacy efforts to increase awareness (…) of (…) joint action at the global, regional and national levels (…).
	4.5. Strengthen financing mechanisms to secure sustained implementation of national and subnational action (…).

### Development of infrastructures to enable active transport

Several actions recommended by the GAPPA ^13^ in the strategic objective “Create active environments” stress the need for governments to develop highly connected mixed-land-use neighborhoods (Action 2.1), to create infrastructures to increase active transport by walking and cycling as a means of PA promotion (Action 2.2) and to improve road safety (Action 2.3).

It will be evident to most readers that these recommendations line up with actions to promote sustainable mobility proposed elsewhere. For example, the UN Economic Commission for Europe has stated that in many nations, more than 30% of final energy (i.e. energy consumed by end users such as households, industry and agriculture) is consumed in transport ^18^. The report concludes that cities striving to become carbon neutral should develop a comprehensive cycling/walking infrastructure and better integrate working, shopping and entertainment opportunities ^18^. Also, the global “C40 Cities Climate Leadership Group” showcases cities around the world that strive to become carbon neutral by supporting active travel ^19^. Cities such as Copenhagen have demonstrated that it is possible to increase the modal share of walking and cycling (currently 45% of all trips) beyond the share of car use (34%) ^19^.

In order to do so, however, more concerted action involving public health policymakers, practitioners and researchers on the one side and urban/transport planners on the other would be needed. Environmental and climate activists are another important asset in taking a strong stance against motorized transport. The GAPPA recommends to use and further develop the WHO Health Economic Assessment Tool for Cycling and Walking (HEAT) ^20^ to enable an assessment of the health, climate and environment-related benefits of transport and urban design policies (Appendix 2 of the GAPPA). On top of that, we would recommend promotion of good practice examples of cities that are already shifting from motorized traffic to active travel such as Copenhagen or Amsterdam. The direct public health burden of motorized transport may serve as an important supporting argument: Globally, there are 1.36 million deaths due to road fatalities (26% pedestrians and cyclists) ^21^ and 3.2 million deaths related to ambient air pollution every year ^22^. Scientific models have also shown that the health benefits of switching from car to bicycle for a commute of 5 km/day (one way) are worth about 1,300 € per person and year, while the negative effects of pollution and accidents are much smaller ^23^.

### Green spaces and recreational/exercise facilities to promote PA

Another area of PA promotion with obvious climate connections relates to green spaces and recreational/exercise facilities. The GAPPA addresses these in the context of the strategic objective “Create active environments” (Actions 2.4 and 2.5).

Besides binding carbon dioxide, parks and green spaces play an important role in climate-proofing cities by mitigating urban heat islands ^24^. It has been shown that, in some cities, an additional square kilometer of green space per 1,000 people might prevent up to 7.4 deaths caused by heat ^25^, that parks have an average cooling effect of 0.94 °C ^26^ and that green spaces can directly and positively influence people’s health ^27^. Furthermore, a study has shown that trees and non-tree vegetation have different effects on PA levels of different ethnic populations; however, non-tree vegetation was negatively associated with PA across all population groups ^28^. In these areas, public health and PA promotion objectives to create more green spaces align very closely with objectives for counteracting climate change. Some cities have already taken steps to expand green spaces by planting trees, such as Manchester ^29^, or by increasing inner-city vegetation as an important part of climate adaptation, such as Copenhagen ^30^.

By contrast, the “climate case” for other types of recreational and exercise facilities is far less clear. Our knowledge about the carbon footprints of such sites is currently limited. One study by Boussabaine *et al.* ^31^ has shown that heated indoor-pools have a much higher energy consumption per square meter and year (1,250–1,750 kWh) than indoor gyms (210–350 kWh). Likewise, the use of fertilizers on the turf grass surfaces needed for many outdoor sports (e.g. football and cricket) causes high nitrogen dioxide emissions when compared to regular pastures, making these compounds a substantial contributor to land consumption ^32^.

In order to better align efforts for PA promotion in this area with those for limiting global warming, the GAPPA recommends implementing assessments of public and green open spaces and natural spaces to evaluate health, climate and environmental benefits of urban ecosystems (Appendix 2 of the GAPPA). We suggest that, additionally, experts should engage with the urban planning and transport sectors to ensure that parks and green spaces are built in close proximity to people’s homes and are easily accessible by active and public transport. Regarding recreational and exercise facilities, land-use and carbon footprint need to be considered in the planning of new facilities and the decision to maintain existing ones. One might also need to reconsider whether new outdoor facilities for sports that require vast land use (e.g. golf, baseball) should be built. Generally, facilities that require extensive heating or cooling are likely to have a higher carbon footprint, i.e. indoor facilities will tend to have a more negative impact on the climate than outdoor facilities. Therefore, newly built indoor infrastructures should preferably be multi-purpose and/or be able to contribute to energy production, e.g. via roof-mounted solar panels.

### Physical activity and exercise programs

While many health promoters and researchers are likely to be aware of the potential links between active transport, PA facilities and climate change, other areas of PA promotion may have rarely been considered with respect to their effects on speeding up or slowing down global warming. Importantly, this applies to the GAPPA’s strategic objective “Create active people”: Actions 3.3, 3.4, 3.5 and 3.6 recommend for the implementation of PA programs and services to be tailored to different target groups, to take place across different settings and to be supported in co-development by all stakeholders and grassroots initiatives. This aspect is strongly related to the previous one, as the type of PA and exercise programs that can be offered depends on the availability of green spaces and recreational/exercise facilities.

To our knowledge, there has been very little research on the carbon footprint of different sport and exercise programs. A recent study by Wicker ^33^ has investigated the travel behavior (e.g. for training, league games, day trips) for German athletes in 20 different sports. The results indicate average carbon emissions of 844 kg per person and year, with stark variation across different sports. Most individual sports, such as climbing/bouldering (1,156 kg CO _2_) and surfing (2,074 kg) have higher emissions than team sports such as soccer (337 kg). However, there are also some individual sports (e.g. fitness/gym with 228 kg) with a comparably small carbon footprint. The study found some nature sports, such as alpine skiing, to have a particularly high carbon footprint ^33^. The environmental impact of these sports has also been pointed out by previous studies ^34, 35^.

Future efforts for PA promotion through services and programs should consider carbon emissions. Partly, these emissions are caused by motorized travel to and from such offers, and partly also by the energy consumption of the facilities they take place in. To minimize motorized travel, offers and programs should be easily reachable by active and public transport. Offers should be attractive to multiple target groups, which will help increase local participation and, as a consequence, shorten the distances that teams need to travel for away matches.

To minimize emissions, activities using facilities with comparably low carbon emissions should be promoted with priority (e.g. outdoor Zumba rather than water aerobics in a heated indoor pool). Respecting the seasonality of activities (e.g. winter sports) will certainly help in this regard. If (recreational) league play is involved, strong efforts should be made to limit (motorized) travel. This might be achieved by encouraging tournaments involving multiple teams in a single location rather than round-robin series of individual home/away matches. Where possible, events should be scheduled during daylight hours to avoid the need for artificial lighting.

### Mass communication campaigns and mass participation events

Another core element of many policy recommendations to promote PA, including the GAPPA’s strategic objective “Create active societies”, are campaigns and events. Actions 1.1 and 1.2 recommend communication campaigns to inform the public about the multiple benefits of PA, and action 1.3 suggests community mass events for PA.

Mass communication campaigns that stress the environmental benefits of active transport line up with objectives for the reduction of carbon emissions. Potentially, future campaigns should place even more emphasis on promoting walking and cycling as an important means for health and environmental benefits. Additionally, they should also (where appropriate) include advice on being physically active in hot weather. Even though most studies indicate that temperature has a positive correlation with the PA behavior of children and adults ^36^, and adults have been found not to modify their PA behaviour on days with high temperatures ^37^, this topic is of particular relevance for vulnerable groups. There is evidence for older people, that the weather influences PA patterns ^38^ and that PA in hot weather can have detrimental health effects ^39^. Campaigns should also highlight sporting activities that have a comparably low carbon footprint. Additionally, weather service providers could incorporate alerts about unsafe PA conditions into their products (e.g. heat advisory and excessive heat warning).

By contrast, using mass events as a means of PA promotion might be rather a double-edged sword from an environmental perspective. The high carbon footprint of professional events such as the Football World Cup has already been described ^40^, while their presumed ‘festival effect’ on PA behavior seems to be very limited ^41^. Comparable research on amateur mass events or those in which recreational athletes compete alongside professionals is scarce, but there is reason to believe that events such as major marathons also come with considerable carbon emissions. Most of these emissions will be caused by participants’ (air) travel to the venue – for example, around 47% of finishers of the New York Marathon in the 2010s were international participants ^42^.

Taking this into consideration, mass events at the community level should be within easy reach of public and active transport in order to limit the climate impact. Organizers of such events might also need to balance the number of participants from other regions or nations and potentially even consider downsizing. Additionally, events would need to be organized in a way that limits detrimental environmental effects, e.g. by avoiding plastic-bottled water, reducing overall waste, offering vegetarian/vegan food options and limiting free giveaways with high carbon footprints such as t-shirts. As a source of inspiration, the Canadian Sport Tourism Alliance has issued guidelines on how to host sustainable sport events ^43^.

### Training of professionals

In its strategic objectives “Create active societies” and “Create active people”, the GAPPA advises countries to invest in the training of professionals for PA (Action 1.4), in particular but not limited to (physical education, PE) teachers (Action 3.1) and health professionals (Action 3.2).

Research indicates that professionals’ training on PA in general leaves much room for improvement ^44^, so that cross-references between PA and climate in curricula can be expected to be even less frequent. A look at current training standards for exercise prescription, such as the ACSM Guidelines ^45^, shows that health professionals are provided with information on how to advise patients on PA in hot environments. However, patient advice regarding the environmental benefits of walking and cycling are currently not covered, not to mention guidance regarding activities that come with a comparably low carbon footprint. Implementing such information into future training of health professionals will more closely align PA promotion with objectives for the reduction of greenhouse gas emissions.

By and large, the same can be said to hold true for the training of PE teachers. In many countries, the climate impact of different exercises and sport tourism might not be explicitly covered. For example, the PE curriculum for secondary schools in the German state of Bavaria requires all pupils to learn winter sports ^46^, which is consequently an integral part of the university curriculum for PE teacher education ^47^. Both schools and universities often teach the required competences through practical alpine or cross-country skiing courses in the Alps. Studies predict the closure of many ski resorts in the Alps due to lack of snow unless massive amounts of artificial snow are used on a regular basis ^48^, thus causing high carbon emissions ^35^. Consequently, re-considering the types of sports and exercise that are taught as part of PE in schools (and by extension in PE teacher training) might be warranted. Preferably, these should be sports/exercises that can be performed locally or regionally, have a low carbon footprint and require little land-use.

## Implications for future PA promotion

Our cursory analysis has demonstrated a considerable potential for alignment between strategies for PA promotion and efforts for the reduction of greenhouse gas emissions. The GAPPA acknowledges the importance of this topic by referring to the SDG on climate action. However, the link between climate action and PA promoting policies/interventions remains unspecific as it is limited to a few sentences. Our analysis shows that these links are already widely acknowledged in some action areas of the GAPPA, most notably in the field of active transport (strategic objective “create active societies”) and regarding green spaces and (at least in part) recreational/exercise facilities (strategic objective “create active environments”). In other areas, such as media campaigns, mass events and professional training, however, the alignment of PA promotion with efforts to reduce carbon dioxide emissions could still be improved.

In order to actually give PA promotion a new, more climate-conscious outlook, changes will be required in PA policymaking, research and surveillance As policy actions, the GAPPA proposes health economic assessments of health, climate and environmental benefits in the areas of active transport and urban design (Action 2.1) and public and green open spaces and natural spaces (Action 2.4). However, these recommendations are just mentioned in the appendix and are not an adequate substitute for a more systematic look at climate-conscious policy development, research and surveillance. The GAPPA’s strategic objective “Create Active Systems” that targets the governance of PA promotion (without linking it to climate change) served as a basis for our following recommendations (Actions 4.1-4.5).

### Climate-conscious policy development for PA promotion

For one, it will be important to raise awareness for the potential benefits and hazards of sport and PA for the climate among policymakers across all levels of government, the media, the private sector and community leaders (Action 4.4). This does not only pertain to the fields of health and sport but also to other relevant sectors such as transport, environment, urban design, tourism and social care. The transport sector, in particular, has a huge potential for devising policies that combine increased health/quality of life with a reduction in carbon dioxide emissions. Environmental protection groups and climate initiatives should also be considered as potential allies for PA promotion.

As also advocated by the GAPPA (Action 4.1), a logical next step is to increase the integration of the two issues in policy frameworks, leadership structures and governance systems, e.g. via multisectoral coordination mechanisms. Historically, both the health and the environmental sector have called for mainstreaming their concerns into all sectors of government – one in the form of “Health in all policies” ^49^, the other under the moniker of “Environmental policy integration” ^50^. In practice, however, there still seems to be room for improvement: data on the implementation of the EU Council Recommendation for Health-Enhancing PA across Sectors indicate that the transport and environment sector are integrated into cross-sectoral PA coordination mechanisms in only 17 out of 27 participating EU Member States ^51^.

Another important action area (Action 4.5) in this context are financing mechanisms. In the future, it will be important to further integrate health and environment-related funding lines to ensure sustainable financial support for activities that promote both the climate and population-level PA. Yet again, data from the EU indicate that it is difficult for governments to even collect information on investments made in sectors other than health that may also help promote PA, be it as an intended or unintended side effect, let alone to act towards a better coordination of sectoral funding ^52^.

However, the implementation of climate-conscious policies for PA promotion also depends on economic, political and cultural factors. For example, health spending per capita is lower in low- and middle-income countries ^53^. These financial constraints might severely impact the capacity of these countries to implement policies for climate change mitigation in general and for health promotion in particular.

### Climate-conscious PA research and surveillance

Research and surveillance (Actions 4.2 and 4.3) are covered as important cornerstones for future PA policy in the GAPPA. However, with respect to climate change, there are a number of key issues in these areas that should be urgently tackled, including the following:

•    Understanding the environmental impact of various forms of sport and exercise in order to tailor promotion efforts towards those with a low carbon footprint.

•    Identifying ways to limit the carbon footprint of sport tourism.

•    Developing curricula to integrate knowledge on the interconnections of PA promotion and climate protection in the training of health and other professionals.

•    Increasing our understanding of how to succeed in transitioning neighborhoods and communities towards high rates of active transport.

Importantly, current PA surveillance systems should strive to integrate indicators that have a high relevance for climate change. This could mean that physical activity questionnaires should include dedicated measures for walking and biking, thus enabling governments to monitor changes in active transport behavior more accurately. Currently-utilized questionnaires often either only assess walking (such as the International Physical Activity Questionnaire IPAQ, ^54^) or walking and cycling in a combined indicator (Global Physical Activity Questionnaire GPAQ ^55^). A positive exception is the relatively new European Health Interview Survey Physical Activity Questionnaire EHIS ^56^, which does assess walking and biking via separate indicators.

The same holds true for PA policy monitoring. As mentioned above, the regular joint EU/WHO surveys on the implementation of the EU Council Recommendation for Health-Enhancing PA across Sectors ^51, 57^ already provide information on a set of highly useful indicators such as the level of cycling and walking, and other supplementary WHO tools (such as the HEPA Policy Audit Tool ^58^ and the HEAT Tool for the health economic assessment of cycling and walking ^59^) may help countries gather additional data. In the years ahead, efforts to strengthen PA policy monitoring and to potentially integrate it with similar efforts in the field of transport, environmental and climate policy should be stepped up.

### Limitations

As this article focuses mainly on climate change mitigation, it does not cover the broader perspective of climate change adaptation and resilience. Likewise, it does not address other ecological aspects of sustainability, such as the loss of biodiversity. Additionally, greenhouse gas emissions have several impacts on the climate besides rising temperatures, such as an increase in extreme weather events, a change in precipitation patterns and a rise in sea-levels, which are not addressed in our paper. Future research at the interface of PA promotion and climate change could address these aspects.

## Conclusions

There are several interconnections between PA promotion and climate action. While they are most recognizable with regards to active transport, green spaces and – partially – recreational or exercise facilities, these links could be strengthened in other areas (media campaigns, mass events, professional training). Climate-conscious policy development, research and surveillance are needed in the field of PA. Recognizing the close alignment between PA promotion and climate action is an important message for public health professionals and policymakers.

## Data availability

No data are associated with this article.
