# Supplementary material for: Physical activity promotion in the age of climate change
Source: F1000Res. 2020 Nov 20;9:349. Originally published 2020 May 11. [Version 2] doi: 10.12688/f1000research.23764.2 (PMC7883311; doi:10.12688/f1000research.23764.2)
Supplement: Peer review history

# Peer review: Reviewer response for version 2

**DOI:** 10.5256/f1000research.30644.r75123

**Published:** 2021-02-12

**Authors:** Stephanie Levy

**Affiliations:** 1 Department of Anthropology, Hunter College, New York, NY, USA

The authors have addressed all of my comments.

Is the topic of the opinion article discussed accurately in the context of the current literature?

Yes

Are arguments sufficiently supported by evidence from the published literature?

Yes

Are all factual statements correct and adequately supported by citations?

Yes

Are the conclusions drawn balanced and justified on the basis of the presented arguments?

Yes

Reviewer Expertise:

Biological anthropology, human energetics, cardio-metabolic health, climate change

I confirm that I have read this submission and believe that I have an appropriate level of expertise to confirm that it is of an acceptable scientific standard.

# Peer review: Reviewer response for version 2

**DOI:** 10.5256/f1000research.30644.r75122

**Published:** 2020-12-07

**Authors:** Kevin Lanza

**Affiliations:** 1 Michael & Susan Dell Center for Healthy Living at The University of Texas Health Science Center at Houston (UTHealth) School of Public Health in Austin, TX, USA

With revisions, authors have markedly improved the manuscript, which serves as a strong foundation for physical activity promotion in the face of climate change. Inclusion of Table 1 makes for a standalone, cohesive manuscript, and the authors did a fine job stating the focus of this manuscript and its limitations. Organization into subsections permits quick access as a reference document, which I plan to use in future research.

 A final, minor suggestion is to decide whether to change "green space" to "greenspace." See below article for use of "greenspace."

 Taylor, Lucy, and Dieter F. Hochuli. "Defining greenspace: Multiple uses across multiple disciplines." *Landscape and Urban Planning* 158 (2017): 25-38 ^1^.

Is the topic of the opinion article discussed accurately in the context of the current literature?

Yes

Are arguments sufficiently supported by evidence from the published literature?

No

Are all factual statements correct and adequately supported by citations?

Yes

Are the conclusions drawn balanced and justified on the basis of the presented arguments?

Yes

Reviewer Expertise:

climate change; physical activity; health equity; urban heat island adaptation

I confirm that I have read this submission and believe that I have an appropriate level of expertise to confirm that it is of an acceptable scientific standard.

## References

[1] : Defining greenspace: Multiple uses across multiple disciplines. *Landscape and Urban Planning*.2017;158: 10.1016/j.landurbplan.2016.09.024 25-38 10.1016/j.landurbplan.2016.09.024

# Peer review: Reviewer response for version 1

**DOI:** 10.5256/f1000research.26223.r70762

**Published:** 2020-10-16

**Authors:** Stephanie Levy

**Affiliations:** 1 Department of Anthropology, Hunter College, New York, NY, USA

The authors argue that many of action points outlined by the recent Global Action Plan on Physical Activity (GAPPA) can be leveraged to also mitigate carbon emissions and global climate change. In particular, the authors highlight how efforts to promote physical activity by establishing active transport and green spaces can also serve to limit carbon emissions. The article is well researched and the argument is clearly presented. However, the paper would benefit from a discussion of the economic, political, and cultural factors that will constrain stakeholders’ ability to carry out these synergistic policies. For instance, in middle-income countries there is a simultaneous increase in carbon emissions and growing prevalence of sedentary lifestyles; however, the capacity to implement climate-minded GAPPA policies might be more limited due to financial constraints.

Is the topic of the opinion article discussed accurately in the context of the current literature?

Yes

Are arguments sufficiently supported by evidence from the published literature?

Yes

Are all factual statements correct and adequately supported by citations?

Yes

Are the conclusions drawn balanced and justified on the basis of the presented arguments?

Yes

Reviewer Expertise:

Biological anthropology, human energetics, cardio-metabolic health, climate change

I confirm that I have read this submission and believe that I have an appropriate level of expertise to confirm that it is of an acceptable scientific standard, however I have significant reservations, as outlined above.

# Response

**Published:** 2020-11-05

**Authors:** Sven Messing

**Affiliations:** University of Erlangen-Nuremberg, Germany

We would like to thank the reviewer for her valuable feedback. We have addressed her comments by adding a paragraph to the discussion section.

 A1: The authors argue that many of action points outlined by the recent Global Action Plan on Physical Activity (GAPPA) can be leveraged to also mitigate carbon emissions and global climate change. In particular, the authors highlight how efforts to promote physical activity by establishing active transport and green spaces can also serve to limit carbon emissions. The article is well researched and the argument is clearly presented. However, the paper would benefit from a discussion of the economic, political, and cultural factors that will constrain stakeholders’ ability to carry out these synergistic policies. For instance, in middle-income countries there is a simultaneous increase in carbon emissions and growing prevalence of sedentary lifestyles; however, the capacity to implement climate-minded GAPPA policies might be more limited due to financial constraints.

 Q1: Thank you very much for pointing out this important aspect. We have added it to the discussion by including the following paragraph at the end of the section: “Climate-conscious policy development for PA promotion” that refers to this issue:

 “However, the implementation of climate-conscious policies for PA promotion also depends on economic, political and cultural factors. For example, health spending per capita is lower in low- and middle-income countries. These financial constraints might severely impact the capacity of these countries to implement policies for climate change mitigation in general and for health promotion in particular.”

# Peer review: Reviewer response for version 1

**DOI:** 10.5256/f1000research.26223.r63281

**Published:** 2020-05-21

**Authors:** Kevin Lanza

**Affiliations:** 1 Michael & Susan Dell Center for Healthy Living at The University of Texas Health Science Center at Houston (UTHealth) School of Public Health in Austin, TX, USA

In this timely and clearly written article, the authors describe the relation between physical activity promotion and climate change, and do so through the World Health Organization’s Global Action Plan on Physical Activity 2018-2030 (GAPPA). From the four strategic objectives (i.e., create active societies, environments, people, and systems) of GAPPA and each objective’s proposed policy actions, the authors offer five perspectives that connect physical activity to climate change: 1) development of infrastructures to enable active transport, 2) green spaces and recreational/exercise facilities to promote PA, 3) physical activity and exercise programs, 4) mass communication campaigns and mass participation events, and 5) training of professionals. The authors conclude with the implications of their overview for future PA promotion, in particular on climate-conscious 1) policy development for PA promotion and 2) PA research and surveillance.

 I commend the authors for this article because the link between physical activity and climate change has not been adequately addressed in the literature, and there is potential for public health and environmental sectors in local government to partner on the topic. Before sharing my thoughts on specific sections of the article, I have a few overall comments. The article would benefit from a table that includes each of the GAPPA objectives and a summary of each of the policy actions. This way, readers can refer to GAPPA while the authors share their five perspectives. Second, there are several impacts of greenhouse gas emissions on our climate (e.g., rising temperatures, increase in extreme weather events, change in precipitation patterns, and sea-level rise), but the authors seem to address only rising temperatures and its relation to physical activity promotion. Similarly, the authors focus their perspectives on physical activity and climate change mitigation (i.e., opportunities for the reduction of greenhouse gas emissions), yet less attention is given to climate change adaptation and resilience. Lastly, the authors should review their article for minor issues with grammar (e.g., inconsistent use of the Oxford comma, “the GAPPA” versus “GAPPA,” and “build” versus “built”) and language choice (e.g., “extremely well” and “less climate-friendly”).

 Please see below for my comments specific to select sections.

Introduction

 The authors state that there are “… stubbornly high rates of physical inactivity in adolescents and adults…” and “PA rates in children are higher but still leave room for improvement.” Both of these statements can be refined. There is a difference between physical inactivity (i.e., sedentary behavior) and not being sufficiently active (i.e., failing to reach recommended levels of physical activity for health benefits). This distinction is important, because although children may have higher PA rates than adults (which depends on method of surveillance and individual-level factors), children in the US are recommended to engage in more PA each week than adults. The authors can be more precise here, and include values.

Development of infrastructures to enable active transport

 The authors focus their overview of active transport on the reduction of greenhouse gas emissions and the benefits to human health. The authors conclude this section with a statement on the health burden of motorized transport—I am wondering if the authors can dig deeper into the literature here. Can the authors tease out how many road fatalities involve a non-motor vehicle (e.g., pedestrian and bicycle)? Similarly, can the authors cite research that has modeled the amount of lives saved, injuries prevented, and pollution reduced from switching from vehicular transport to alternative modes?

Green spaces and recreational/exercise facilities to promote PA

 The authors focus their overview of green spaces on carbon sequestration, urban heat island mitigation, and climate adaptation; and their overview of recreational/exercise facilities on energy and land consumption. I think this section can be improved by providing further detail on each of the above points. Regarding green spaces, I would mention the amount of cooling offered by parks, which has been found to be 0.94°C, on average:

 Bowler, D. E., Buyung-Ali, L., Knight, T. M., & Pullin, A. S. (2010). Urban greening to cool towns and cities: A systematic review of the empirical evidence. *Landscape and urban planning*, *97*(3), 147-155 ^1^.

 The specific vegetation is also worth mentioning, in relation to how trees and non-tree vegetation have different effects on physical activity levels of different populations; and how climate change is impacting what trees are able to be planted in an area with continued global warming:

 Lanza, K., Stone Jr, B., & Haardörfer, R. (2019). How race, ethnicity, and income moderate the relationship between urban vegetation and physical activity in the United States. *Preventive medicine*, *121*, 55-61 ^2^.

 Lanza, K., & Stone Jr, B. (2016). Climate adaptation in cities: What trees are suitable for urban heat management?. *Landscape and Urban Planning*, *153*, 74-82 ^3^.

 For recreational/exercise facilities, the authors can mention outdoor fitness classes/groups/meet-ups, which do not have the energy use requirements (e.g., air conditioning and lighting) of indoor facilities.

Physical activity and exercise programs

 The authors focus their overview of exercise programs on the reduction of greenhouse gas emissions and the benefits to human health. The authors state “…outdoor facilities with comparably low carbon emissions should be promoted with priority (e.g. outdoor Zumba rather than water aerobics in a heated indoor pool).” This recommendation also relates to the authors’ previous perspective on recreational/exercise facilities. In organizing the article’s content, the authors may want to consider green spaces as a separate perspective, and recreational/exercise facilities and exercise programs as another perspective.

Mass communication campaigns and mass participation events

 The authors focus their overview of mass communication and participation on the reduction of greenhouse gas emissions and the benefits to human health. The authors raise the pertinent point that weather conditions may influence physical activity patterns. However, most studies show temperature to have a positive correlation with physical activity, with individuals being more active during summer over other seasons:

 Chan, C. B., & Ryan, D. A. (2009). Assessing the effects of weather conditions on physical activity participation using objective measures. *International journal of environmental research and public health*, *6*(10), 2639-2654 ^4^.

 In recent work, US adults have been found to not modify their physical activity behavior on days with high temperatures, which suggests cities in hot climates may be placing adults at increased risk of exertional heat illness. The authors recommend incorporating the risk of exertional heat illness in health impact assessments and deploying heat management strategies (i.e., tree planting and installation of reflective building materials):

 Lanza, K., Stone, B., Chakalian, P. M., Gronlund, C. J., Hondula, D. M., Larsen, L., ... & Haardörfer, R. (2020). Physical Activity in the Summer Heat: How Hot Weather Moderates the Relationship Between Built Environment Features and Outdoor Physical Activity of Adults. *Journal of Physical Activity and Health*, *1*(aop), 1-9 ^5^.

 The authors point about the environmental impact of sports apparel production seems tangential to physical activity promotion and climate change, and therefore may be removed. Instead, I believe this article section would benefit from including content on the increased risk of exertional heat illness from projected temperature increases. The authors could also include content on how weather service providers, such as the National Weather Service in the United States, can incorporate alerts about unsafe physical activity conditions within its heat products (e.g., heat advisory and excessive heat warning).

Is the topic of the opinion article discussed accurately in the context of the current literature?

Yes

Are arguments sufficiently supported by evidence from the published literature?

No

Are all factual statements correct and adequately supported by citations?

Yes

Are the conclusions drawn balanced and justified on the basis of the presented arguments?

Yes

Reviewer Expertise:

climate change; physical activity; health equity; urban heat island adaptation

I confirm that I have read this submission and believe that I have an appropriate level of expertise to confirm that it is of an acceptable scientific standard, however I have significant reservations, as outlined above.

## References

[1] : Urban greening to cool towns and cities: A systematic review of the empirical evidence. *Landscape and Urban Planning*.2010;97(3) : 10.1016/j.landurbplan.2010.05.006 147-155 10.1016/j.landurbplan.2010.05.006

[2] : How race, ethnicity, and income moderate the relationship between urban vegetation and physical activity in the United States. *Preventive Medicine*.2019;121: 10.1016/j.ypmed.2019.01.022 55-61 10.1016/j.ypmed.2019.01.022 30707905

[3] : Climate adaptation in cities: What trees are suitable for urban heat management?. *Landscape and Urban Planning*.2016;153: 10.1016/j.landurbplan.2015.12.002 74-82 10.1016/j.landurbplan.2015.12.002

[4] : Assessing the Effects of Weather Conditions on Physical Activity Participation Using Objective Measures. *International Journal of Environmental Research and Public Health*.2009;6(10) : 10.3390/ijerph6102639 2639-2654 10.3390/ijerph6102639 20054460PMC2790098

[5] : Physical Activity in the Summer Heat: How Hot Weather Moderates the Relationship Between Built Environment Features and Outdoor Physical Activity of Adults. *Journal of Physical Activity and Health*.2020;17(3) : 10.1123/jpah.2019-0399 261-269 10.1123/jpah.2019-0399 31918409

# Response

**Published:** 2020-11-05

**Authors:** Sven Messing

**Affiliations:** University of Erlangen-Nuremberg, Germany

We would like to thank the reviewer for his valuable feedback. We have addressed his comments by modifying our article in the respective sections as described below.

 Q1: In this timely and clearly written article, the authors describe the relation between physical activity promotion and climate change, and do so through the World Health Organization’s Global Action Plan on Physical Activity 2018-2030 (GAPPA). From the four strategic objectives (i.e., create active societies, environments, people, and systems) of GAPPA and each objective’s proposed policy actions, the authors offer five perspectives that connect physical activity to climate change: 1) development of infrastructures to enable active transport, 2) green spaces and recreational/exercise facilities to promote PA, 3) physical activity and exercise programs, 4) mass communication campaigns and mass participation events, and 5) training of professionals. The authors conclude with the implications of their overview for future PA promotion, in particular on climate-conscious 1) policy development for PA promotion and 2) PA research and surveillance.

 I commend the authors for this article because the link between physical activity and climate change has not been adequately addressed in the literature, and there is potential for public health and environmental sectors in local government to partner on the topic. Before sharing my thoughts on specific sections of the article, I have a few overall comments. The article would benefit from a table that includes each of the GAPPA objectives and a summary of each of the policy actions. This way, readers can refer to GAPPA while the authors share their five perspectives. Second, there are several impacts of greenhouse gas emissions on our climate (e.g., rising temperatures, increase in extreme weather events, change in precipitation patterns, and sea-level rise), but the authors seem to address only rising temperatures and its relation to physical activity promotion. Similarly, the authors focus their perspectives on physical activity and climate change mitigation (i.e., opportunities for the reduction of greenhouse gas emissions), yet less attention is given to climate change adaptation and resilience. Lastly, the authors should review their article for minor issues with grammar (e.g., inconsistent use of the Oxford comma, “the GAPPA” versus “GAPPA,” and “build” versus “built”) and language choice (e.g., “extremely well” and “less climate-friendly”).

 Please see below for my comments specific to select sections.

 A1: Thank you very much for your positive comment. The link between physical activity and climate change has indeed a high potential for joint action of the public health and environmental sector.

 As suggested, we added a table that provides an overview about all GAPPA objectives and policy actions (table 1). Furthermore, we added a sentence at the end of the introduction to clarify that the article focuses on climate change mitigation and the relation of rising temperatures and physical activity promotion. Additionally, we described this focus as a limitation in the discussion section and recommended future research to address these aspects. The minor issues with grammar and language choice were addressed as well.

 Introduction

 Q2: The authors state that there are “… stubbornly high rates of physical inactivity in adolescents and adults…” and “PA rates in children are higher but still leave room for improvement.” Both of these statements can be refined. There is a difference between physical inactivity (i.e., sedentary behavior) and not being sufficiently active (i.e., failing to reach recommended levels of physical activity for health benefits). This distinction is important, because although children may have higher PA rates than adults (which depends on method of surveillance and individual-level factors), children in the US are recommended to engage in more PA each week than adults. The authors can be more precise here, and include values.

 A2: Thank you very much for this helpful comment. We have refined the first statement and included values. We state now that there are “… stubbornly high rates of insufficient physical activity in adolescents (81,0%) and adults (27,5%). …”. We removed the second statement.

 Development of infrastructures to enable active transport

 Q3: The authors focus their overview of active transport on the reduction of greenhouse gas emissions and the benefits to human health. The authors conclude this section with a statement on the health burden of motorized transport—I am wondering if the authors can dig deeper into the literature here. Can the authors tease out how many road fatalities involve a non-motor vehicle (e.g., pedestrian and bicycle)? Similarly, can the authors cite research that has modelled the amount of lives saved, injuries prevented, and pollution reduced from switching from vehicular transport to alternative modes?

 A3: We agree with this suggestion and added additional information on the number of deaths per year due to road fatalities among pedestrians and cyclists (26% of 1.36 million) and on the health benefits of shifting from car to bicycling (1,300 Euro per person and year for a commute of 5 km per day, effects of pollution and accidents are much smaller).

 Green spaces and recreational/exercise facilities to promote PA

 Q4: The authors focus their overview of green spaces on carbon sequestration, urban heat island mitigation, and climate adaptation; and their overview of recreational/exercise facilities on energy and land consumption. I think this section can be improved by providing further detail on each of the above points. Regarding green spaces, I would mention the amount of cooling offered by parks, which has been found to be 0.94°C, on average:

 Bowler, D. E., Buyung-Ali, L., Knight, T. M., & Pullin, A. S. (2010). Urban greening to cool towns and cities: A systematic review of the empirical evidence. Landscape and urban planning, 97(3), 147-1551.

 The specific vegetation is also worth mentioning, in relation to how trees and non-tree vegetation have different effects on physical activity levels of different populations; and how climate change is impacting what trees are able to be planted in an area with continued global warming:

 Lanza, K., Stone Jr, B., & Haardörfer, R. (2019). How race, ethnicity, and income moderate the relationship between urban vegetation and physical activity in the United States. Preventive medicine, 121, 55-612.

 Lanza, K., & Stone Jr, B. (2016). Climate adaptation in cities: What trees are suitable for urban heat management?. Landscape and Urban Planning, 153, 74-823.

 For recreational/exercise facilities, the authors can mention outdoor fitness classes/groups/meet-ups, which do not have the energy use requirements (e.g., air conditioning and lighting) of indoor facilities.

 A4: We added additional information regarding the cooling effect of parks (Bowler et al 2010) and the influence of trees on physical activity levels (Lanza et al 2019). This evidence strengthens the argument and is highly appreciated.

 We did not, however, add the study that analyses what trees are suitable for urban heat management because we felt that it is not closely related to our line of argument. Outdoor fitness classes are mentioned in the next section (physical activity and exercise programs).

 Physical activity and exercise programs

 Q5: The authors focus their overview of exercise programs on the reduction of greenhouse gas emissions and the benefits to human health. The authors state “…outdoor facilities with comparably low carbon emissions should be promoted with priority (e.g. outdoor Zumba rather than water aerobics in a heated indoor pool).” This recommendation also relates to the authors’ previous perspective on recreational/exercise facilities. In organizing the article’s content, the authors may want to consider green spaces as a separate perspective, and recreational/exercise facilities and exercise programs as another perspective.

 A5: We added the following sentence to the first paragraph of this section: “This aspect is strongly related to the previous one, as the type of PA and exercise programs that can be offered depends on the availability of green spaces and recreational/exercise facilities.”

 We decided not to reorganize the article’s overarching structure because we believe that this would not have solved the issue completely: Physical activity and exercise programs usually take place either in green spaces or in recreational/exercise facilities. There is an overlap between the GAPPA’s strategic objectives “create active environments” and “create active people”.

 Mass communication campaigns and mass participation events

 Q6: The authors focus their overview of mass communication and participation on the reduction of greenhouse gas emissions and the benefits to human health. The authors raise the pertinent point that weather conditions may influence physical activity patterns. However, most studies show temperature to have a positive correlation with physical activity, with individuals being more active during summer over other seasons:

 Chan, C. B., & Ryan, D. A. (2009). Assessing the effects of weather conditions on physical activity participation using objective measures. International journal of environmental research and public health, 6(10), 2639-26544.

 In recent work, US adults have been found to not modify their physical activity behavior on days with high temperatures, which suggests cities in hot climates may be placing adults at increased risk of exertional heat illness. The authors recommend incorporating the risk of exertional heat illness in health impact assessments and deploying heat management strategies (i.e., tree planting and installation of reflective building materials):

 Lanza, K., Stone, B., Chakalian, P. M., Gronlund, C. J., Hondula, D. M., Larsen, L., ... & Haardörfer, R. (2020). Physical Activity in the Summer Heat: How Hot Weather Moderates the Relationship Between Built Environment Features and Outdoor Physical Activity of Adults. Journal of Physical Activity and Health, 1(aop), 1-95.

 A6: We clarified this section and added both references: “Even though most studies indicate that temperature has a positive correlation with the PA behavior of children and adults, and adults have been found not to modify their PA behaviour on days with high temperatures, this topic is of particular relevance for vulnerable groups.  There is evidence for older people, that the weather influences PA patterns and that PA in hot weather can have detrimental health effects”.

 Q7: The authors point about the environmental impact of sports apparel production seems tangential to physical activity promotion and climate change, and therefore may be removed. Instead, I believe this article section would benefit from including content on the increased risk of exertional heat illness from projected temperature increases. The authors could also include content on how weather service providers, such as the National Weather Service in the United States, can incorporate alerts about unsafe physical activity conditions within its heat products (e.g., heat advisory and excessive heat warning).

 A7: We deleted the sentence about the environmental impact of sports apparel production as it is not closely related to our topic. As suggested, we added a sentence about the incorporation of alerts about unsafe PA conditions into the products of weather service providers.
